# Effect of mobile-assisted education on health promoting lifestyle and blood sugar of women with gestational diabetes: a randomised controlled trial

**DOI:** 10.1136/bmjnph-2023-000802

**Published:** 2023-12-06

**Authors:** Maryam Maleki, Parvaneh Mousavi, Parvin Abedi, Dariush Rokhafrooz, Elham Maraghi

**Affiliations:** 1 Departmentof Midwifery, School of Nursing and Midwifery, Diabetes Research Center, Ahvaz Jundishapur University of Medical Sciences, Ahvaz, Iran (the Islamic Republic of); 2 Instructor of Midwifery, School of Nursing and Midwifery, Diabetes Research Center, Ahvaz Jundishapur University of Medical Sciences, Ahvaz, Khuzestan, Iran (the Islamic Republic of); 3 Midwifery Department, Menopause Andropause Research Center, Ahvaz Jundishapur University of Medical Sciences, Ahvaz, Iran (the Islamic Republic of); 4 Medical Education, Nursing Department, Ahvaz Jundishapur University of Medical Sciences, Ahvaz, Iran (the Islamic Republic of); 5 Biostatistics Department, Ahvaz Jundishapur University of Medical Sciences, Ahvaz, Iran (the Islamic Republic of)

**Keywords:** Gestational diabetes, Mental health, Nutrition assessment

## Abstract

**Background:**

The gestational diabetes causes complications for the mother and the baby.

**Methods:**

It was a randomised controlled trial that was conducted in Public Health Center No 1 in Baghmalek, Khuzestan province, Iran. Seventy-six women with gestational diabetes mellitus (GDM) were recruited and randomly allocated into an intervention (n=38) and a control group (n=38). A mobile app was developed, and the content of the educational programme was designed according to the six dimensions of Health Promotion Lifestyle Profile (HPLP). Participants in the intervention group followed instructions about healthy lifestyle for 4 weeks, whereas the control group received only routine care. A demographic questionnaire, and the HPLP-II were used to gather the data.

Health promoting lifestyle and blood sugar of participants were measured after 4 weeks.

**Results:**

The total score of HPLP was 98.34±13.99 and 92.39±14.56 before the intervention in intervention and control groups, respectively, which was improved significantly in the intervention group compared with the control group after intervention (143.13±23.41 vs 100.39±16.69, p<0.0001). Also, the scores of stress management, responsibility, interpersonal relationship, spiritual growth, nutrition and physical activity were significantly improved in the intervention group. Fasting blood sugar and blood sugar 2 hours after meal significantly reduced in the intervention group compared with the control group (86.05±7.71 mg/dL vs 93.92±5.52 mg/dL) and (113.65±10.96 mg/dL vs 124.97±9.15 mg/dL), (p=0.001), respectively.

**Conclusions:**

Our results showed that offering educational programmes based on mobile apps can improve healthy lifestyle and blood sugar in women with GDM.

**Trial registration number:**

IRCT20200817048434N1

**Website address:**

https://www.irct.ir/search/result?query=IRCT20200817048434N1

**WHAT IS ALREADY KNOWN ON THIS TOPIC**

- Treatment of gestational diabetes mellitus (GDM) is primarily based on diet and physical activity for cases with mild abnormality in blood sugar, while for those with more serious abnormalities, medications such as metformin or insulin are recommended.

**WHAT THIS STUDY ADDS**

- Our results showed that offering educational programmes based on mobile apps can improve healthy lifestyle and blood sugar in women with GDM.

**HOW THIS STUDY MIGHT AFFECT RESEARCH, PRACTICE OR POLICY**

- Health policy makers can use an education programme using mobile app to improve the health of women with gestational diabetes.

## Introduction

Gestational diabetes mellitus (GDM) is defined as any glucose intolerance first recognised in pregnancy.^1^ According to the International Association of Diabetes in Pregnancy Study Group (IADPSG), the global prevalence of GDM is 14%, with a regional prevalence of 7% in North America, 10.4% in Europe and 27.6% in the Middle East and North Africa.^2^ The prevalence of GDM in Iran which is 3.41%, with the highest and lowest prevalence of 18.6% and 1.3%, respectively.^3^

GDM has maternal and fetal consequences. For example, women affected with GDM are in a greater risk of pre-eclampsia, caesarean section and birth canal trauma due to macrosomia.^4^ If GDM happens at the time of fetal organogenesis, the rate of congenital abnormalities and abortion will increase.^5^ Fetal macrosomia, shoulder dystocia and hypoglycaemia at birth are some neonatal short-term consequences. In addition, childhood and adulthood obesity, increased risk of diabetes in later life and cardiometabolic disorders are some examples of long-term consequences of GDM.^6^ Preconception screening tests for glucose intolerance and counselling should be considered as preventive measures.^7 8^

Treatment of GDM is primarily based on diet and physical activity for cases with mild abnormality in blood sugar, while for those with more serious abnormalities, medications such as metformin or insulin are recommended. Evidence shows that around 85% of women with GDM respond to lifestyle change.^9^ Proper diet including essential macronutrients and micronutrients, which both allow normal fetal growth and prevent the maternal obesity, has outmost importance.^10^ Education of pregnant women plays an important role in management of GDM. It can improve the compliance of women with lifestyle change or medication and decrease maternal and neonatal consequences of GDM.^11^ Although, education is now incorporated in the management of GDM in all public health centres in Iran, the compliance of women with recommendation is not high. A study in Iran showed that compliance of pregnant GDM women with the treatment regimens is low and only half of women could control their diabetes.^12^ At the present time, education about changing lifestyle is done face to face and individually in public health centres of Iran. The method of education is an important factor, which does not have variety in Iran’s health centre. On the other hand, education using application has been recognised to be useful in self-care improvement in other diseases such as prevention of COVID-19 infection, or to improving self-care of women affected with pre-eclampsia during COVID-19 pandemic.^13 14^ However, there is limited information regarding using e-health and mobile app in controlling GDM.

### Objectives

The primary aim of the current study was to evaluate the effect of education through mobile app on health promoting lifestyle of women with gestational diabetes. The secondary aim was to assess the effect of this intervention on blood sugar.

## Methods

This was a parallel randomised controlled trial on 76 women with gestational diabetes who were randomly allocated into an intervention group receiving educational content through mobile app (n=38) and a control group (n=38).

### Inclusion/exclusion criteria

Women were eligible to participate in this study if they: aged 18–35, had gestational diabetes (fasting blood sugar, FBS>92 mg/dL and abnormal 2 hour fasting glucose tolerance test using 75 g glucose>153 mg/dL),^15^ were at gestational age of 24–28 weeks (in this gestational age, the screening for gestational diabetes is done), had basic literacy, had a smart phone, were gravida 1 or 2, and received low or moderate scores from Health Promoting Lifestyle Questionnaire (HPLQ) (scores<103 and 104–155 from HPLQ). Women were excluded from the study if they had diabetes that needed medication or were affected with pre-eclampsia (having proteinuria and blood pressure >140/90 mm Hg) or hypertension (blood pressure>140/90 mmHg).

### Sample size

Using a previous study,^16^ assuming α=0.05, β=95%, d=11.14 and s=15.12, and using the following formula, we calculated the sample size to be n=31 in each group. We added 20% for attrition, and the final sample size became 38 in each group.

$n=\frac{2{\left(1-\alpha /2+z_{\beta }\right)}^{2}s^{2}}{d^{2}}$

### Setting

All women with GDM who were admitted to the Public Health Center No 1 in Baghmalek, Khuzestan province, Iran were considered for sampling. Those who met the inclusion criteria were recruited in this study. All blood tests were done in the laboratory affiliated to this health centre. Data collection was started in January 2021 and completed in April 2021.

### Randomisation and allocation concealment

The eligible women were randomly allocated into two groups of intervention and control using block randomisation with a block size of 4 and an allocation ratio of 1:1. Letters A and B were considered for the intervention and control groups, respectively. Using computer software (https://www.sealedenvelope.com/simple-randomiser/v1/lists), 76 participants were randomly allocated into two groups of intervention and control.

Codes allocated to each participant were written on a piece of paper and kept with the clerk of the public health centre. Therefore, prior to the commencement of intervention, neither the researchers nor the participants knew about the participant grouping. Due to the nature of the study, blinding was not possible.

### Instruments

A demographic and obstetric information questionnaire (including questions about age, gravidity, number of children, gestational age, body mass index, educational attainment, occupation and intended pregnancy) was used for gathering data. The results of blood sugar tests were recorded in a checklist. The content validity was used to check the validity of demographic questionnaire and the checklist, by this way that 10 faculty members reviewed and confirmed the questions.

Furthermore, the Health Promotion Lifestyle Profile (HPLP-II) was used for gathering data about lifestyle of women. This questionnaire has 52 questions in 6 dimensions including spiritual growth and self-actualisation (11 questions), responsibility for health (13 questions), interpersonal relationships (8 questions), stress management (6 questions), sports and physical activities (7 questions) and nutrition (7 questions). The questionnaire was scored based on a 4-items Likert scale (never=1, sometimes=2, often=3 and always=4). The minimum score is 52 and the maximum is 208. The total score is categorised into three levels: a score less than 103 represents a poor lifestyle, 104–155 indicates an average lifestyle and a score of ≥156 shows a good lifestyle. Walker *et al* conducted a study to check the psychometric properties of HPLP-II. They first conducted content validity using the experts’ opinion and then conducted a construct validity using factor analysis, that confirmed the six dimensions of the questionnaire. The internal consistency was confirmed by Cronbach’s alpha (0.943). Also test–retest evaluation was measured to be 0.892.^17^ The psychometric evaluation of the Persian version of this questionnaire was done by Mohammadi-zeidi *et al* in Iran.^18^ In the Persian version, three questions were removed because of low factor load, and the number of questions reduced to 49 ((spiritual growth and self-actualisation (9 questions), responsibility for health (13 questions), interpersonal relationships (7 questions), stress management (5 questions) and nutrition (7 questions)).

### Development of mobile APP

The mobile phone app was developed by one of the researchers (DR) as explained in the following. First, the content of the teaching material was determined according to six dimensions of HPLP including (nutrition and diet, physical activity and exercise, responsibility, interpersonal relationship, spiritual growth and stress management). The content of educational programme was prepared by the research team using guidelines of Iranian Ministry of Health and literature review. The validity of this programme was checked by 10 faculty members.

The development of the mobile app was done in three parts namely predevelopment, development and postlunch. First, the objectives were determined to answer two questions: (1) What do you want to achieve at the end? (2) What problems does this application solve?

For these purposes, the opinions of midwifery faculty members and research team regarding goals, content, user style, characteristics of audience and the implementation platform of the application were determined. Then, the characteristics of the designable application were determined, and a wireframe (wireframe is a layout of a mobile phone application) was stablished. The native Iranian platforms (JOAPE) was used to design the application. The mobile application was designed using coding and multimedia programme for the mobile operating system Android 4 and above. At first, the application was evaluated by the research team several times regarding its feasibility. Then, the application was sent to 10 faculty members of the Midwifery Department, and its flaws were removed. Finally, the final version was used for implementation in the intervention group. One of the researchers (MM) installed the application on the mobiles of participants in the intervention group. The participants used the programme in the presence of the researcher and make sure that it worked properly.

### Intervention

The educational application was included eight parts as follows: gestational diabetes, nutrition, physical posture, exercise, advice, contact with us and other programmes.

The first section included information about gestational diabetes, its definition, symptoms and consequences, and ways of monitoring blood sugar. In the second section, the user is provided with information about increasing patients’ awareness of the importance of following a diet, choosing the right foods, recommendations for healthy cooking, suggestions for meals and snacks, different methods to get more fruits and vegetables, foods that diabetic patients should avoid and ways to deal with overeating.

The third section dealt with proper posture habits while standing, correct sitting posture, how to sit on the floor, sleeping on one side, getting up from sleep and picking up objects.

In the fourth section that was dedicated to exercise and physical activity, the user was provided with information about increasing patients' knowledge of the health benefits of regular physical activity, types of suitable physical activities during pregnancy, the amount of exercise recommended per day, the benefits of walking and the correct walking techniques. The fifth section was concerned with ways of and recommendations for increasing spiritual growth, improving interpersonal relationships, increasing responsibility and stress management.

In the sixth section, the ways of contact with the researcher and software designer in case of any problem in the software or questions about how to use the software were explained. Recommendations were introduced in the seventh section, and other related programmes were introduced in the eighth section. It should be noted that in order for a better understanding of the educational materials, attractive and relevant images were used in each section.

After the software was installed on the mobile phone of the mothers in the intervention group, the intervention was started.

Also, the researcher followed the participants in the intervention group at least twice a week through phone calls or text messages, and if there was any ambiguity, the necessary explanations were provided for a better understanding of the educational items. In addition, in order to better follow-up and control the mothers, a group was formed in WhatsApp by the researcher. Also, the contact number of the researcher was provided to the participants from the very beginning of the intervention, so that they could call privately and solve their problems.

For the control group, routine healthcare was provided by a midwife. By this way that a midwife provided the necessary training about reducing carbohydrate consumption and regular physical activity. The blood sugar then measured 4 weeks after these recommendations.

### Outcomes

Primary outcomes: health promoting lifestyle

Secondary outcomes: blood sugar.

### Follow-up

FBS and blood sugar 2 hours after meal were measured at the beginning of the study and 4 weeks after intervention (all measurements were reported in milligram (mg) per decilitr (dL)). The participants were asked to attend a reference laboratory for measuring FBS and 2 hour blood sugar after meal. Also, participants were asked to complete HPLP questionnaires at the beginning of the study and 4 weeks thereafter.

### Statistics

Data analysis was done using SPSS V.20. The quantitative variables were reported using mean and SD, and qualitative variables, using frequency (percentage). The normality of quantitative variables was assessed by the Shapiro-Wilk test. The comparison of qualitative variables in the two groups was done using χ^2^ or Fisher’s exact tests. Comparison of the quantitative variables between two groups was performed using the independent samples t-test or its non-parametric equivalent (Mann-Whitney test). Effect of intervention on post-test outcome measures was examined using analysis of covariance (ANCOVA), adjusting for respondent’s age, parity, job status and pretest scores. The significance level of the tests was considered as 0.05.

## Results

**Figure 1:**
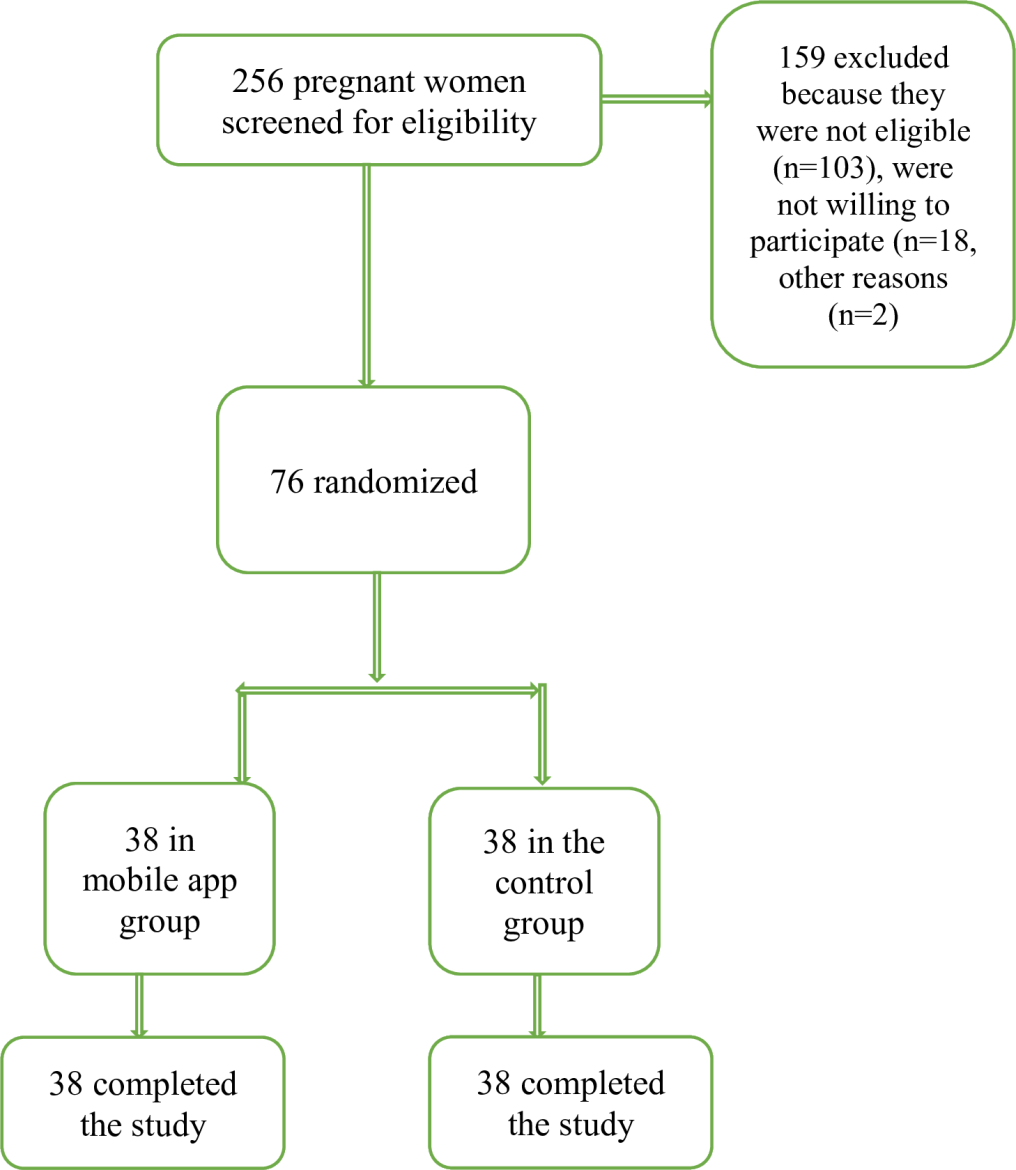
Flow-diagram of recruitment and retention of participants in the study.

In this study, we enrolled 76 women with GDM in the intervention control group (n=38 each). None of participants withdrew from the study (figure 1). Table 1 shows the demographic and obstetric characteristics of the participants. As this table shows, the mean±SD age of women was 30.47±3.13 and 28.92±2.44 years in the intervention and control groups, respectively (p=0.019). Most of the women in the intervention (63.2%) and in the control (52.6%) groups were nulliparous, had high school or diploma education and were housewives. Two groups showed significant difference regarding age, parity and occupation.

**Table 1:** Demographic and obstetric characteristics of participants in two groups of intervention and control

Variables	Intervention, n=38	Control, n=38	P value
	Mean (SD)		
Age (years)	30.47±3.13	28.92±2.44	0.019
Gestational age (weeks)	26.47±0.86	26.81±1.13	0.824
Body mass index (kg/m^2^)	23.58±2.55	23.46±2.61	0.843
	N (%)		
Gravidity			
Nulliparous	24 (63.2)	20 (52.6)	0.028
1	8 (21.1)	17 (44.7)	
≤2	6 (15.8)	1 (2.6)	
Education			
High school	17 (44.7)	13 (34.2)	0.428
Diploma	15 (39.5)	18 (47.4)	
University education	6 (15.8)	7 (18.4)	
Occupation			
Employed	3 (7.9)	13 (34.2)	0.010
Housewife	35 (92.1)	25 (65.8)	
Type of pregnancy			
Intended	34 (89.47)	32 (84.2)	0.736
Not-intended	4 (10.95)	6 (15.8)	

Table 2 shows the scores of HPLP and its dimensions in intervention and control groups. As evident from this table, after intervention, the score of stress management was significantly improved in the intervention group compared with the control group (15.02±2.58 vs 10.2±07.01, p<0.0001). The score of responsibility was 25.18±4.39 in the intervention group, which improved to 37.94±6.84 after intervention. However, this score experienced no significant improvement in the control group. This score was statistically different between the two groups (p<0.0001). The score of interpersonal relationship was 15.68±3.65 before intervention in the intervention group, which improved to 20.94±3.27 after intervention, while in the control group, it remained almost unchanged (from 14.44±2.91 to 14.39±3.42). The two groups showed significant differences regarding this score (p<0.0001). As far as the score of spiritual growth before intervention was concerned, the two groups did not show any significant difference (22.42±4.78 vs 20.18±5.34, p>0.05), while after intervention, they were significantly different in this regard (30.4±10.12 vs 21.73±4.71, p<0.0001). Participants in the intervention group could improve their nutrition and physical activity after intervention compared with the control group (p<0.0001). The total score of HPLP in the intervention and control groups was 98.34±13.99 and 92.39±14.56, respectively, before intervention, that means that both groups had poor lifestyle. This score improved significantly in the intervention group (143.13±23.41) compared with the control group (100.39±16.69) (p<0.0001). This means that the lifestyle of the intervention group improved to a moderate type, while in the control group remained poor.

**Table 2:** Descriptive statistics and ANCOVA* results for HPLP and its dimensions in two groups of intervention and control

Outcome	Intervention, n=38	Control, n=38	F	P value	Effect size^†^
Stress management					
Before	11.18±3.46	10.05±1.69	60.71	<0.0001	0.46
After	15.02±2.58	10.07±2.01			
Responsibility					
Before	25.63±4.99	25.18±4.39	56.64	<0.0001	0.45
After	37.94±6.84	26.60±5.27			
Interpersonal relationships					
Before	15.68±3.65	14.44±2.91	45.28	<0.0001	0.39
After	20.94±3.27	14.39±3.42			
Spiritual growth					
Before	22.42±4.78	20.18±5.34	52.47	<0.0001	0.43
After	30.4±10.12	21.73±4.71			
Nutrition					
Before	12.68±1.899	12.65±2.36	60.93	<0.0001	0.46
After	21.86±3.30	15.73±3.57			
Physical activity					
Before	10.71±2.14	10.73±2.07	61.16	<0.0001	0.47
After	18.02±5.09	11.78±2.20			
Total score					
Before	98.34±13.99	92.89±14.56	63.61	<0.0001	0.48
After	143.13±23.41	100.39±16.69			

*Adjustment is considered for respondent’s age, parity, job status and baseline measures of the outcomes.

†Partial Eta Squared.

ANCOVA, analysis of covariance.

**Figure 2:**
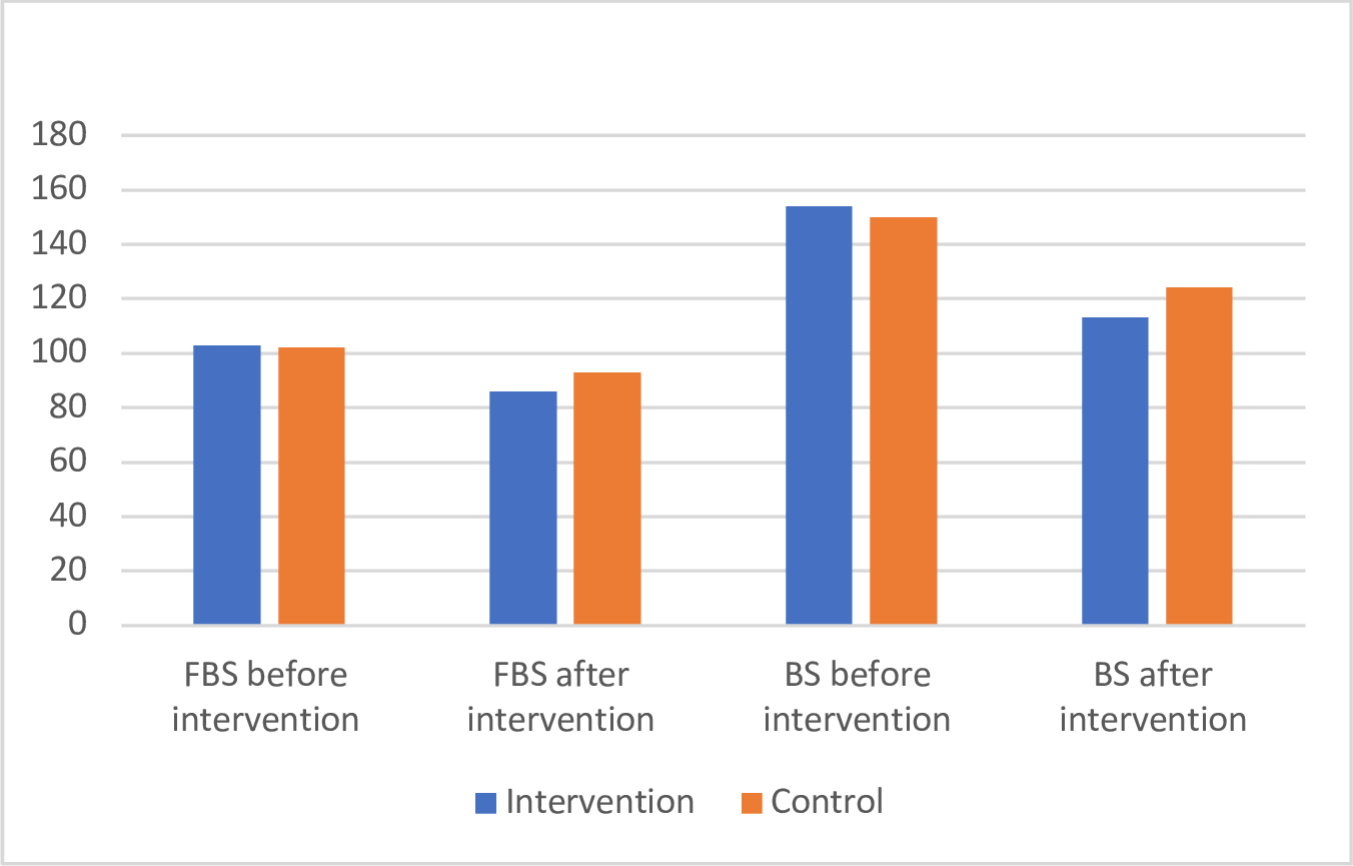
FBS and blood sugar 2 hours after meal (BS) in the intervention and control groups before and after intervention. FBS, fasting blood sugar.

Table 3 and figure 2 show the FBS and blood sugar 2 hours after meal before and after intervention in the two groups. FBS was 103.86±9.10 mg/dL and 102.84±10.45 mg/dL before intervention in the two groups of intervention and control, respectively. Although FBS decreased in both groups, the reduction was more significant in the intervention group (86.05±7.71 mg/dL vs 93.92±5.52 mg/dL, p<0.0001). Before intervention, the blood sugar 2 hours after meal was 154.23±8.86 mg/dL and 150.94±11.68 mg/dL in the intervention and control groups, respectively. After intervention, the reduction of this parameter was more significant in the intervention group compared with control group (113.65±10.96 mg/dL vs 124.97±9.15 mg/dL, p<0.0001). In other word, the level of FBS and blood sugar 2 hour after meal returned to normal levels after 4 weeks in the intervention group.

**Table 3:** Descriptive statistics and ANCOVA^*^ results for the fasting blood sugar and blood sugar 2 hours after meal in two groups of intervention and control

Outcome	Intervention, n=38	Control, n=38	F	P value	Effect size^†^
FBS; mg/dL					
Before	103.86±9.10	102.84±10.45	22.86	<0.0001	0.25
After	86.05±7.71	93.92±5.52			
Blood sugar 2 hours after meal; mg/dL					
Before	154.23±8.86	150.94±11.68	35.67	<0.0001	0.34
After	113.65±10.96	124.97±9.15			

A milligram is one-thousandth of a gram.

A deciliter measures fluid volume that is 1/10 litre.

*Adjustment is considered for respondent’s age, parity, job status and baseline measures of the outcomes.

†Partial η^2^.

ANCOVA, analysis of covariance; FBS, fasting blood sugar.

## Discussion

This study was designed to evaluate the effect of mobile-assisted education on health promoting lifestyle and blood sugar of women with GDM. Our results showed that the scores of all dimensions of health promoting lifestyle and stress management significantly improved in the intervention group compared with the control group. The total score of High Pressure Processing improved from poor to moderate in the intervention group, while it remained at a poor level in the control group.

Evidence shows that there is a relationship between stress and GDM, and women with GDM have more oxidative stress compared with their otherwise healthy counterparts.^19^ In line with our results, a systematic review by Gilbert *et al* including 16 studies found that lifestyle changes focusing on diet and physical activity can improve the lifestyle of pregnant women with GDM and decrease their stress.^20^

Our results also indicated a significant improvement in the score of responsibility in the intervention group compared with the control group. According to a systematic review by Igwesi-Chidobe *et al*,^21^ including 27 studies, all interventions such as self-management programmes, diet, physical activity and vitamin and mineral supplementation were effective in prevention and treatment of GDM.

The results of the present study showed that after intervention, there was a significant improvement in the scores of interpersonal relationship, and spiritual growth in the intervention group compared with the control group. There is evidence in the literature showing that intervention in terms of spiritual growth and faith could improve GDM, but the underlying mechanisms are not clear.^22^ In a quasi-experimental study on 40 pregnant women with GDM, Azari *et al* 23 found that eight sessions of spiritual group therapy could significantly decrease the anxiety and improve the quality of life of women compared with the control group, which is consistent with our results.

We also found that participants in the intervention group could improve their nutrition and physical activity, as well as their total score of HPLP after intervention compared with the control group. These findings are in line with Simmons *et al* study^24^ on 436 women with GDM who were randomised in four groups of healthy eating, physical activity, both healthy eating and physical activity, and control. They found that weight gain of women in the healthy eating and physical activity group was less than of women in the other groups. Also, another randomised controlled trial by Karimipour *et al* 25 on 60 pregnant women with GDM found that healthy lifestyle recommendations could significantly improve diet and physical activity in the intervention group compared with the control group.

The results of the present study showed that FBS and blood sugar 2 hours after meal decreased significantly in the intervention group compared with the control group. In other word, the level of FBS and blood sugar 2 hour after meal returned to normal levels after 4 weeks in the intervention group.

Although these two parameters decreased in the control group, the reduction was more significant in the intervention group. In line with our study, a previous study on 665 women with GDM showed that most women controlled their blood sugar following a particular diet, especially low carbohydrate diet.^26^ Sagastume *et al* 27 found that intervention based on diet and physical activity for at least 6 months among women with GDM and type 2 diabetes could significantly reduce the level of HBA1c, FBS and blood sugar 2 hours after meal, which confirms our results.

### Strengths and limitations of the study

This was the first study in Iran to use an educational programme based on a mobile application for improving the healthy lifestyle and blood sugar of women with GDM. Despite its strengths, this study has some limitations. First, data about compliance with healthy lifestyle were gathered from the participants, which might have been affected by recall bias. Second, this study was conducted in one public health centre in Baghmalek, Khuzestan province, Iran and this may limit generalisability of findings of this study to other population and regions. Third, we recruited 76 women with GDM. Future studies with larger sample size are needed to confirm the results of this study. Fourth, the participants followed only for 4 weeks. A longer follow-up period could provide insights into the sustainability of the observed changes in health-promoting lifestyle and blood sugar control over time. Fifth, due to the nature of the study, blinding was not feasible. The lack of blinding could introduce potential bias, as both the researchers and participants were aware of group assignments. And finally, while the study measured health-promoting lifestyle and blood sugar levels, other relevant health indicators such as changes in body mass index, maternal and fetal outcomes, were not addressed.

## Conclusion

The results of the present study showed that using an educational programme based on a mobile application can improve healthy lifestyle, and blood sugar in women with gestational diabetes. Health policymakers are therefore recommended to consider different methods other than traditional educational methods for changing lifestyle in women with GDM.

10.1136/bmjnph-2023-000802.supp1 Supplementary data

10.1136/bmjnph-2023-000802.supp2 Supplementary data

## Data Availability

All data relevant to the study are included in the article or uploaded as supplementary information. The data of this study will be available upon the reasonable request from corresponding author.
