# Supplementary material for: Effect of mobile-assisted education on health promoting lifestyle and blood sugar of women with gestational diabetes: a randomised controlled trial
Source: BMJ Nutr Prev Health. 2023 Dec 6;6(2):310–7. doi: 10.1136/bmjnph-2023-000802 (PMC11009530; doi:10.1136/bmjnph-2023-000802)
Supplement: Supplementary data [file bmjnph-2023-000802supp002.pdf]

Protocol of the study

The effect of mobile-assisted education on health promoting lifestyle and blood sugar of women with gestational diabetes: A randomized controlled trial

General information
Protocol identifying number: **IRCT20200817048434N1**
Name of funder: Ahvaz Jundishapur University of Medical Sciences
Name and contact details of the investigators: Maryam Maleki, Parvaneh Mousavi, Parvin Abedi, Dariush Rokhafrooz, Elham Maraghi

Responsibilities of each investigator: All authors were involved in the conception of the study. Dariush Rokhafrooz will develop the mobile app. Maryam Maleki will collect the data. Parvaneh Mousavi and Parvin Abedi will supervise the research process. Elham Maraghi will analyze the data. All authors will be responsible for data interpretation. Parvin Abedi will be responsible for writing the first draft of the manuscript.

Name and address of the clinical laboratory: The laboratory of Public Health Center No 1 in Baghmalek, Iran

2. Rational and background information:

Gestational diabetes mellitus (GDM) is defined as any glucose intolerance first recognized in pregnancy [1]. According to the International Association of Diabetes in Pregnancy Study Group (IADPSG), the global prevalence of GDM is 14%, with a regional prevalence of 7% in North America, 10.4% in Europe, and 27.6% in the Middle East and North Africa [2]. According to a systematic review including 24 studies and 1011 participants, the prevalence of GDM in Iran which is located in the Middle East was 3.41%, with the highest and lowest prevalence of 18.6% and 1.3%, respectively [3].

GDM has maternal and fetal consequences. For example, women affected with GDM are in a greater risk of pre-eclampsia, cesarean section, and birth canal trauma due to macrosomia [4]. If GDM happens at the time of fetal organogenesis, the rate of congenital abnormalities and abortion will increase [5]. Fetal macrosomia, shoulder dystocia, and hypoglycemia at birth are some neonatal short-term consequences. In addition, childhood and adulthood obesity, increased risk of diabetes in later life, and cardiometabolic disorders are some examples of long-term consequences of GDM [6]. Pre-conception screening tests for glucose intolerance and counseling should be considered as preventive measures [7].

Education of pregnant women plays an important role in management of GDM. It can improve the compliance of women with lifestyle change or medication and decrease maternal and neonatal consequences of GDM [8].

3. Study objectives: The primary aim of the current study is to evaluate the effect of education through mobile app on lifestyle of women with gestational diabetes, and the secondary aim is to assess the effect of this intervention on blood sugar.

4. Study design: This study will be a parallel randomized controlled trial.

Inclusion/exclusion criteria: Women will be eligible to participate in this study if they: have gestational diabetes (high FBS and abnormal two-hour fasting glucose tolerance test using 75 gr glucose), are at gestational age of 24-28 wks, have basic literacy, have a smart phone, are gravida 1 or 2, and will receive low or moderate scores from Health Promoting Lifestyle Questionnaire. Women will be excluded from the study if they have overt diabetes or will be affected with pre-eclampsia or hypertension. We expect that data collection will take 1 year.

Setting: The pregnant women with GDM who will attend to the Public Health Center No 1 in Baghmalek, Khuzestan province, Iran will be recruited according to the inclusion criteria. The laboratory of this health center will be used for all blood tests.

4. Methodology

Instruments: A demographic and obstetric information questionnaire (including questions about age, gravidity, number of children, gestational age, body mass index, educational attainment, occupation, and intended pregnancy) will be used for gathering data. The content validity of this questionnaire will be confirmed. The results of blood sugar tests will be recorded in a checklist. Furthermore, the Health Promotion Lifestyle Profile (HPLP-II) will be used for gathering data about lifestyle of women. This questionnaire has 52 questions in 6 dimensions including spiritual growth and self-actualization (11 questions), responsibility for health (13 questions), interpersonal relationships (8 questions), stress management (6 questions), sports and physical activities (7 questions) and nutrition (7 questions). The questionnaire is scored based on a 4-items Likert scale (never=1, sometimes=2, often=3, and always=4). The minimum score is 52 and the maximum is 208. The total score is categorized into three levels: a score less than 103 represents a poor lifestyle, 104-155 indicates an average lifestyle, and a score of ≥156 shows a good lifestyle [9]. The psychometric evaluation of the Persian version of this questionnaire was done by Mohammadi-zeidi et al [10]. In the Persian version, three questions were removed because of low factor load, and the number of questions reduced to 49.

Intervention: The mobile phone app will be developed by one of the researchers (DR) as following: First, the content of the teaching material will be determined according to six dimensions of HPLP including (nutrition and diet, physical activity and exercise, responsibility, interpersonal relationship, spiritual growth, and stress management). The mobile application will

be designed using coding and multimedia program for the mobile operating system Android 4 and above. The application will be evaluated by the research team. For the pilot study, the alpha version of the application will be sent to 10 faculty members of the Midwifery Department, and its flaws will be removed. Finally, the beta version will be used for implementation in the intervention group. The educational application will be included 8 sections as follows: gestational diabetes, nutrition, physical posture, exercise, advice, contact with us, and other programs.

The first section will be included information about gestational diabetes, its definition, symptoms and consequences, and ways of monitoring blood sugar. In the second section, the user will be provided with information about increasing patients' awareness of the importance of following a diet, choosing the right foods, recommendations for healthy cooking, suggestions for meals and snacks, different methods to get more fruits and vegetables, foods that diabetic patients should avoid, and ways to deal with overeating.

The third section will be dealt with proper posture habits while standing, correct sitting posture, how to sit on the floor, sleeping on one side, getting up from sleep, and picking up objects.

In the fourth section that will be dedicated to exercise and physical activity, the user will be provided with information about increasing patients' knowledge of the health benefits of regular physical activity, types of suitable physical activities during pregnancy, the amount of exercise recommended per day, the benefits of walking, and the correct walking techniques. The fifth section will be concerned with ways of and recommendations for increasing spiritual growth, improving interpersonal relationships, increasing responsibility, and stress management.

In the sixth section, the ways of contact with the researcher and software designer in case of any problem in the software or questions about how to use the software will be explained.

Recommendations will be introduced in the seventh sections, and other related programs will be introduced in the eighth section. It should be noted that in order for a better understanding of the educational materials, attractive and relevant images will be used in each section.

After the software will be installed on the mobile phone of the mothers in the intervention group, the intervention will be started.

Also, the researcher will follow the participants in the intervention group at least twice a week through phone calls or text messages, and if there will any ambiguity, the necessary explanations will be provided for a better understanding of the educational items. In addition, in order to better follow up and control the mothers, a group will be formed in WhatsApp by the researcher. Also, the contact number of the researcher will be provided to the participants from the very beginning of the intervention, so that they can call privately and solve their problems.

For the control group, routine health care will be provided by a midwife. In Iranian health centers, educational pamphlets are provided for mothers with diabetes, and necessary health recommendations regarding diet, regular physical activity to control blood sugar will be made.

6. **Safety considerations**: All blood tests will be collected in the laboratory of Health center No 1 in Baghmalek, Iran, by a trained nurse.

7. Follow-up: Fasting blood sugar (FBS) and blood sugar 2 hours after meal will be measured at the beginning of the study and 4 weeks after intervention. Also, participants will be asked to complete HPLP questionnaires at the beginning of the study and four weeks thereafter.

8. Data management and statistical analysis: All data will be analyzed using SPSS version 25. The Chi-square test (or Fisher's Exact test), and independent t-test (or Mann-Whitney test) will be used for categorical and continuous data, respectively. ANCOVA will be used for comparing

blood sugar and HPLP between two groups controlling for baseline. $P<0.05$ will be considered statistically significant.

9. Quality assurance: All laboratory tests will be done in a reference laboratory. Diagnosis of GDM will be done according to high fasting blood sugar and abnormal two-hour fasting glucose tolerance test using 75 gr glucose.

10. Expected outcomes of the study: Primary outcomes: Health promoting lifestyle

Secondary outcomes: Blood sugar

11. Dissemination of results and publication policy: The results of the study will be published in a scientific international journal. Also, the summary of the study will be accessible for all pregnant women in a simple language.

12. Duration of the project: Sampling is expected to take six months. Data analysing, interpretation, and writing the paper needs six months.

13. Anticipated problems: Some women may refuse to attend the clinic due to the COVID-19 pandemic. In this regard, face masks will be distributed free of charge to the participants and social distancing will be maintained.

14. Project management: All authors were involved in the conception of the study. Dariush Rokhafrooz will develop the mobile app. Maryam Maleki will collect the data. Parvaneh Mousavi received the expenses. Parvaneh Mousavi and Parvin Abedi will supervise the research process. Elham Maraghi will analyze the data. All authors will be responsible for data interpretation. Parvin Abedi will be responsible for writing the first draft of the manuscript.

15. Ethics: This research will be performed in line with the principles of the Declaration of Helsinki. Approval was granted by the Ethics Committee of Ahvaz Jundishapur University of Medical Sciences (Ref. No: IR.AJUMS.REC.1399.076). Also, the protocol was registered in

the Iranian Registry for Clinical Trials (Ref. No: IRCT20200817048434N1). Written informed consent will be obtained from eligible women.

16. Budget: Ahvaz Jundishapur University of Medical Sciences will provide the expenses of the research.

17. Supplementary support for the project: For this project, we anticipated that except for expenses that receive from Ahvaz Jundishapur University of Medical Sciences, we will not receive any fees from anywhere else.

18. Collaboration with other researchers of institutions: All researchers in this project are affiliated with Ahvaz Jundishapur University of Medical Sciences and we do not anticipate collaborating with other researchers in other institutions.

References

1.American Diabetes Association. Diagnosis and classification of diabetes. (2014). Diabetes Care, 37(Suppl 1): S81–90.

2. Wang H, Li N, Chivese T, Simmons D, Yang X. IDF diabetes atlas: estimation of global and regional gestational diabetes mellitus prevalence for 2021 by International Association of Diabetes in Pregnancy Study Group Criteria. Diabetes Res Clin Pract. 2022; 183: 109050.

3. Jafari-Shobeiri M, Ghojazadeh M, Azami-Aghdash S, Naghavi-Behzad M, Piri R, Pourali-Akbar Y, et al. Prevalence and Risk Factors of Gestational Diabetes in Iran: A Systematic Review and Meta-Analysis. Iran J Public Health. 2015; 44:1036-44. PMID: 26587467; PMCID: PMC4645723.

4. Mitanchez D, Yzydorczyk C, Siddeek B, Boubred F, Benahmed M, Simeoni U. The offspring of the diabetic mother–short- and long-term implications. *Best Pract Res Clin Obstet Gynaecol.* 2015;29:256–269.

5. Standards of medical care in diabetes—2016: summary of revisions. *Diabetes Care.* 2016; 39 Suppl 1:S4–S5.

6. Murray SR, Reynolds RM. Short-and long-term outcomes of gestational diabetes and its treatment on fetal development. Prenat Diagn. 2020;40: 1085-1091.

7. Dahiya K. New FIGO guidelines on gestational diabetes. Obstet Gynecol. 2017; 129 (5): s170. DOI: 10.1097/01.AOG.0000514097.03289.82

8. Staynova R, Yanachkova V. Improving gestational diabetes management through patient education. Open access peer-reviewed chapter. 2021; DOI: 10.5772/intechopen.100562

9. Walker S, Hill-Polerecky D. (1997). Psychometric evaluation of the health-promoting lifestyle profile II. Uni Neb Med Center.1997; 1:32-4.

10. Mohammadi Zeidi I, Pakpour Hajiagha A, Mohammadi Zeidi B. Reliability and Validity of Persian Version of the Health-Promoting Lifestyle Profile. J Maz Univ Med. 2011; 20:102-13.
